# Metabolic changes during prostate cancer development and progression

**DOI:** 10.1007/s00432-022-04371-w

**Published:** 2022-09-23

**Authors:** Alicia-Marie K. Beier, Martin Puhr, Matthias B. Stope, Christian Thomas, Holger H. H. Erb

**Affiliations:** 1 Department of Urology, Technische Universität Dresden, Fetscherstr. 74, 01307 Dresden, Germany; 2 Department of Urology, Mildred Scheel Early Career Center, Medical Faculty and University Hospital Carl Gustav Carus, Technische Universität Dresden, 01307 Dresden, Germany; 3 Department of Urology, Medical University of Innsbruck, Innsbruck, Austria; 4 Department of Gynecology and Gynecological Oncology, University Hospital Bonn, Bonn, Germany; 5 National Center for Tumor Diseases Partner Site Dresden and German Cancer Center Heidelberg, Heidelberg, Germany; 6 German Cancer Consortium (DKTK), Partner Site Dresden, Dresden and German Cancer Research Center (DKFZ), Heidelberg, Germany

**Keywords:** Metabolic reprogramming, Glutamine, CRPC

## Abstract

Metabolic reprogramming has been recognised as a hallmark in solid tumours. Malignant modification of the tumour’s bioenergetics provides energy for tumour growth and progression. Otto Warburg first reported these metabolic and biochemical changes in 1927. In prostate cancer (PCa) epithelial cells, the tumour metabolism also changes during development and progress. These alterations are partly driven by the androgen receptor, the key regulator in PCa development, progress, and survival. In contrast to other epithelial cells of different entities, glycolytic metabolism in prostate cells sustains physiological citrate secretion in the normal prostatic epithelium. In the early stages of PCa, citrate is utilised to power oxidative phosphorylation and fuel lipogenesis, enabling tumour growth and progression. In advanced and incurable castration-resistant PCa, a metabolic shift towards choline, amino acid, and glycolytic metabolism fueling tumour growth and progression has been described. Therefore, even if the metabolic changes are not fully understood, the altered metabolism during tumour progression may provide opportunities for novel therapeutic strategies, especially in advanced PCa stages. This review focuses on the main differences in PCa’s metabolism during tumourigenesis and progression highlighting glutamine’s role in PCa.

## Introduction

Cellular metabolism in living organisms is a series of chemical reactions that maintain life. It entails complex biochemical processes of specific nutrients, e.g., carbohydrates, fatty acids, and amino acids, to conserve energy homeostasis and macromolecular synthesis. Thus, cellular metabolism can be divided into anabolism (polymerisation of complex macromolecules), catabolism (degradation of molecules to release energy), and waste disposal (elimination of toxic waste from anabolism and catabolism). These processes maintain cell growth, proliferation, and survival (Zhu and Thompson 2019). Several studies have shed new light on how cancer cells reprogram their cellular metabolism, fuelling cell proliferation in the last decades.

**Fig. 1:**
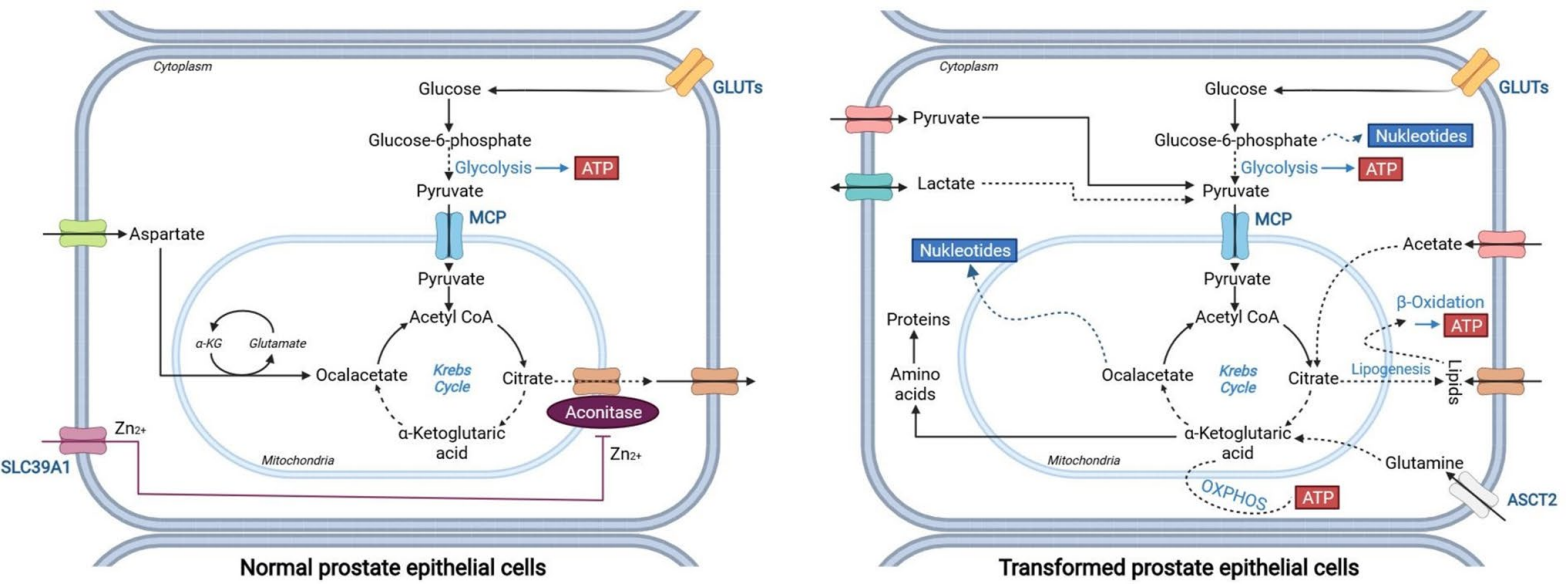
Metabolism of benign (left) versus malignant prostate epithelial cells (right). Left: normal prostate epithelium assimilates glucose and aspartate to be able to produce citrate in large amounts and excrete it for ejaculate. Active zinc transport from the extracellular areas is responsible for the high citrate as it causes inhibition of the aconitase. This inhibition interrupts the Krebs cycle and prevents the conversion of citrate to isocitrate. Consequently, the central ATP generation in normal prostate epithelial cells consists mainly of glycolysis. Right: in PCa cells, metabolic reprogramming is characterised by increased oxidative phosphorylation (OXPHOS) and lipogenesis. As zinc levels are lowered in malignant PCa cells, the inhibition of aconitase is absent and the Krebs cycle is active. As a result, citrate is oxidised to isocitrate or used as a substrate for lipogenesis. In addition, PCa cells take up many exogenous metabolites (e.g., pyruvate, acetate, and glutamine) to maintain the Krebs cycle via anaplerotic reactions. Solid arrows stand for single metabolic steps and dashed arrows for simplified multistep processes. SCL39A1, ASCT2, GLUTs, and MCTs are zinc, glutamine, glucose, and pyruvate transporters, respectively

This review examines the role of metabolic reprogramming in prostate cancer (PCa, Fig. 1). We highlight the main metabolic changes in epithelial prostate cells and PCa cells and concentrate on the role of glutamine in the metabolic processes of PCa.

### The metabolism in benign prostate epithelial cells

Concerning reproduction, the prostate gland plays an essential role as part of the male sexual apparatus. The gland produces a slightly acidic secretion (pH 6.4–6.8), representing 15–30% of the seminal fluid (Fair and Cordonnier 1977; Owen and Katz 2005). The secretion contains phospholipids, cholesterol, fibrinogenase, fibrinolysin, zinc, high citrate levels, and several proteins. The prostatic secretion’s protein content is less than 1% and includes proteolytic enzymes, such as the prostate-specific antigen (kallikrein 3, PSA) (Owen and Katz 2005). In addition, the secretion supplies energy, stabilises, and protects the sperm from the female immune system and vaginal secretions with an acidic pH value of 3.8 to 4.4. Therefore, the benign prostate’s epithelial cell’s metabolism (Fig. 1 left panel) supports sperm functionality by the active production and secretion of high citrate levels (Costello and Franklin 1997). It is suggested that normal prostate epithelial cells have a reprogrammed Krebs cycle (a.k.a. citric acid cycle or tricarboxylic acid cycle) and therefore do not oxidise the produced citrate like most normal cells of different tissue (Costello and Franklin 1997). To this end, prostate cells accumulate high mitochondrial zinc (Zn2+) levels by transporting Zn2+ via the SLC39A1 transporter from the extracellular areas. Zn2+ inhibits the mitochondrial enzyme (m)-aconitase, responsible for citrate oxidation. The accumulating citrate is subsequently secreted into the prostatic fluid. This Krebs cycle reprogramming makes the prostate epithelium unique in the human body, reducing ATP production by the Krebs cycle to 35% compared to citrate-oxidising cells. This reduced ATP production is mainly fuelled by anaplerotic molecules, such as acetate, lipids, and amino acids. They, therefore, allow sparing of pyruvate for the citrate synthesis and excretion and thus maintenance of the Krebs cycle (Costello and Franklin 1997).

### Metabolic changes in primary PCa epithelial cells

Due to the uncontrolled growth and proliferation of malignant cells require a high level of nutrients for cell division, maintenance of tumour cells, and tumour volume expansion (Hanahan and Weinberg 2011; Pavlova et al. 2022). During malignant transformation, PCa cells acquire the ability to oxidise citrate, increasing ATP production (Costello and Franklin 1997; Singh et al. 2006). This metabolic change (Fig. 1 right panel) is accompanied by the reactivation of m-aconitase, which initiates citrate oxidation via the Krebs cycle. As Zn2+ inhibits the enzyme, the reactivation is triggered by lower zinc levels and alterations in the mitochondrial (mt)DNA (Costello and Franklin 1997; Petros et al. 2005; Singh et al. 2006). Moreover, in contrast to other solid tumours, several studies have revealed that the metabolism of primary PCa is highly lipogenic, less glycolytic, and dependent on oxidative phosphorylation (OXPHOS) (Bader et al. 2019; Sadeghi et al. 2015; Scaglia et al. 2021). This unique tumour metabolism is partly driven by the AR-regulated mitochondrial pyruvate carrier (MPC) (Bader et al. 2019). MPC is located at the inner mitochondrial membrane and is responsible for importing pyruvate from the glycolysis into the mitochondrial matrix to incorporate into the Krebs cycle. This pyruvate-filled Krebs cycle is what differentiates PCa metabolism from other solid cancers. In general, it is believed that cancer cells divert pyruvate to fuel other anabolic processes or convert it to lactate (Vaupel and Multhoff 2021). The dependency on pyruvate was validated from expression studies, revealing an increase in pyruvate and succinate-related pathways for utilisation in primary PCa (Bader et al. 2019; Schöpf et al. 2020). Next to the regulation of the MPC, AR also regulates other central metabolic pathways, including glycolysis and lipogenesis, promoting PCa proliferation (Audet-Walsh et al. 2017; Swinnen et al. 1996; Tsouko et al. 2014). However, unlike other solid tumours, 18F-fluorodeoxyglucose (FDG) uptake assays revealed that primary PCa shows little FDG uptake, suggesting that metabolic substrates other than glucose might be required to meet PCa’s anabolic demands (Liu et al. 2001).

Another early event during prostate tumorigenesis and PCa progression is the reprogramming of lipid metabolism (including fatty acids, phospholipids, and cholesterol) (Scaglia et al. 2021). Lipogenesis generates intracellular signalling molecules and resources to produce lipid bilayers and biochemical energy through β-oxidation (Scaglia et al. 2021). Several oncogenic signalling pathways, such as PI3K/AKT and HER2, are suggested to increase lipogenic enzyme expression de novo lipogenesis (DNL) in PCa cells (Scaglia et al. 2021). In addition, increased fatty acid oxidation also serves as a primary energy source. This hypothesis is supported by the overexpression of alpha-methylacyl-CoA racemase (AMACR) in PCa, an enzyme independent of the AR signalling (Scaglia et al. 2021). The enzyme converts (2R)-methylacyl-CoA esters to their (2S)-methylacyl-CoA epimers. Known AMACR substrates are coenzyme A esters and cholesterol-delivered bile acids (Lloyd et al. 2008).

### Aetiology, disease progression, and treatment options of PCa

According to the latest global cancer statistics, PCa is the second most common cancer in men, with 1,276,106 new diagnoses annually and 358,989 deaths worldwide (Ferlay et al. 2021; Sung et al. 2021). Over the years, several risk factors for PCa have been identified. Next to age, ethnicity, genetic predisposition, obesity, and dietary factors are risk factors for developing aggressive PCa (Tan and Naylor 2022). A high-fat diet and reduced vitamin consumption (carotenoids, retinoids, vitamins C, D, and E), minerals (calcium, selenium), and phytoestrogens (isoflavonoids, flavonoids, lignans) lead to an increase in PCa risk (Oczkowski et al. 2021). Moreover, a meta-analysis suggested a connection between pre-existing diabetes and a worse prognosis for men with PCa (Cai et al. 2015; Lee et al. 2016). However, the exact role of cellular metabolism and PCa aggressiveness and progress is not deciphered yet.

PCa treatment depends on the tumour’s stage, patient’s age, overall health, and tumour risk assessment (Cornford et al. 2021; Mottet et al. 2021). Radical prostatectomy or radiotherapy treats localised PCa with a curative approach (Mottet et al. 2021). The 5-year survival rate for localised PCa is ~ 99% (Siegel et al. 2020). However, around 30–40% of the patients experience recurrence (Hennequin et al. 2017; Palermo et al. 2016). If the PCa has already metastasised, drug therapy is the treatment of choice with palliative intention (Cornford et al. 2021). As the development and progress of PCa are highly dependent on androgen receptor (AR) signalling, the golden standard for metastasised PCa treatment is androgen deprivation therapy (ADT) (Obinata et al. 2020). The AR belongs to the steroid hormone receptors and is activated by binding androgenic hormones, e.g., testosterone and dihydrotestosterone (DHT) (Obinata et al. 2020). ADT aims to diminish the activity of the AR axis by either reducing the androgen concentration in the body (medical castration) or directly inactivating the AR using the so-called antiandrogens (e.g., Bicalutamide, Enzalutamide, Apalutamide, or Darolutamide) (Cornford et al. 2021). Despite the favourable initial response of the tumour to ADT, tumour progression occurs again after approximal 2 to 3 years, and the incurable castration-resistant PCa (CRPC) develops (Cornford et al. 2021). CRPC can be treated with taxan-based chemotherapy (Docetaxel or Cabazitaxel), antiandrogens, PARP inhibitors (Olaparib), or radio-ligand therapy (177Lu-PSMA-617) alone or in combination with androgen deprivation (Cornford et al. 2021). However, the 5-year survival rate for patients with metastatic PCa and CRPC is only ~ 30% (Obinata et al. 2020). Therefore, a better understanding of the biology of metastasised PCa, such as metabolism, is necessary to develop new therapeutic strategies.

### The influence of ADT on PCa metabolism

AR signalling is the primary therapeutic target in metastasised PCa. To this end, the AR is indirectly targeted by ligand deprivation or directly targeted by the so-called antiandrogens, such as enzalutamide, apalutamide, or darolutamide (Cornford et al. 2021). In recent decades, AR has been shown to affect growth-promoting and anti-apoptotic genes controlling various metabolic processes. These include multiple energy and biomass supply pathways, such as glycolysis, mitochondrial respiration, metabolism of fatty acids, nucleotides, amino acids, and polyamines (Uo et al. 2020). Therefore, glucose and amino acid membrane transporters, such as the glucose transporter (GLUT)1, GLUT2, and the L-type amino acid transporter 1, controlling import and export processes are regulated in their expression by the AR (Wang et al. 2020; White et al. 2018; Xu et al. 2016). Moreover, the AR regulates the expression of several enzymes involved in metabolic processes. These processes include the AMACR, cytochrome P450 proteins, lactate dehydrogenase A, fatty acid synthase, and hexokinase 2 (Audet-Walsh et al. 2017; Han et al. 2018; Sun et al. 2021b). Therefore, the tumour metabolism is indirectly targeted by directly targeting the AR signalling during the therapy approach of metastasised PCa. Thereby, AR inhibition reduces metabolic processes, and the PCa cells lack the energy to maintain vital signalling pathways.

### Metabolic changes in CRPC cells

Following ADT, 60–70% of patients will progress to a CRPC status within 2–3 years. Heterogeneous mechanisms have been identified in CRPC development, including AR gene amplification, AR overexpression, and mutations leading to consecutive activity or promiscuity, androgen-independent AR activation by outlaw pathways, coactivator overexpression, intratumoral de novo androgen synthesis, and AR loss (Scaglia et al. 2021). Next to the changes in AR signalling, loss of p53 and PTEN expression, MYC amplification, change in expression and activity of STAT proteins, elevated GR expression, and mutations in PI3K, BRAC1, as well as BRAC2, are common in CRPC (Ebersbach et al. 2021; Erb et al. 2020; Scaglia et al. 2021). These alterations have been linked to metabolic reprogramming in several cancers, including PCa (Schiliro and Firestein 2021; Xu et al. 2019, 2021; Zheng et al. 2020). This metabolic reprogramming is accompanied by heterogenic genomic and transcriptomic alterations leading to high CRPC metabolic heterogeneity. Nuclear magnetic resonance analysis of benign prostate hyperplasia, hormone-sensitive PCa, and CRPC patients revealed a metabolic shift in CRPC towards choline, amino acid, and glycolytic metabolism (Zheng et al. 2020). Sun J and colleagues could reveal similar metabolic alterations in human and murine CPRC models (Sun et al. 2021a). The group used [U-13C]glucose or [U-13C]glutamine (Gln) uptake assays to demonstrate altered glycolysis, glutaminolysis, Krebs cycle metabolism, and glutathione redox capacity.

Moreover, the reactivated AR signalling in CRPC increases the expression of the glucose transporters GLUT1, increasing intracellular glucose concentrations and driving glucose metabolism (Wang et al. 2020). These findings suggest that due to multiple metabolic alterations, the Warburg effect does become a more significant metabolic route in CRPC. One phenomenon that the Warburg effect has often accompanied is the support of Gln in the mitochondrial oxidative metabolism as an anaplerotic reaction, filling the Krebs cycle instead of pyruvate (Hensley et al. 2013). Subsequently, the cancer cell’s growth is supported by Gln, one of the critical metabolic characteristics of CRPC (Kaushik et al. 2014; Sun et al. 2021a). In CRPC, Gln consumption is increased, and so is its utilisation in several metabolic processes such as glutaminolysis, Gln anaplerosis into the Krebs cycle, and glutathione synthesis. However, as Gln only support PCa growth, the malignancy is not addicted to Gln as other solid tumours are. You will find a detailed description of the role of Gln in PCa below in the chapter “The glutamine metabolism in PCa”.

In CRPC and other cancers, lipids have also been an essential energy source, contributing to membrane building and acting as a secondary messenger for molecular pathways (Scaglia et al. 2021). As the AR regulate genes involved in the DNL, DNL is suppressed during ADT (Ettinger et al. 2004; Han et al. 2018). However, it is reactivated after the expression of constitutively active AR splice variants in CRPC, consequently increasing genes involved in DNL (Lounis et al. 2020). One key enzyme in DNL is the fatty acid synthase (FASN), which catalyses palmitate synthesis from malonyl-CoA and acetyl-CoA (Scaglia et al. 2021). Therefore, the expression of FASN and other enzymes in the lipogenic pathway is elevated in CRPC, suggesting a role in tumour growth and progress (Ettinger et al. 2004; Migita et al. 2009). Next to its role in DNL, FASN and other steroidogenic enzymes are involved in the intratumoral synthesis of androgens, such as testosterone from cholesterol or other steroid precursors in CRPC (Montgomery et al. 2008). Therefore, the intratumoral testosterone synthesis leads to an activation of AR-target genes and maintains tumour cell survival (Scaglia et al. 2021).

### The glutamine metabolism in PCa

**Fig. 2:**
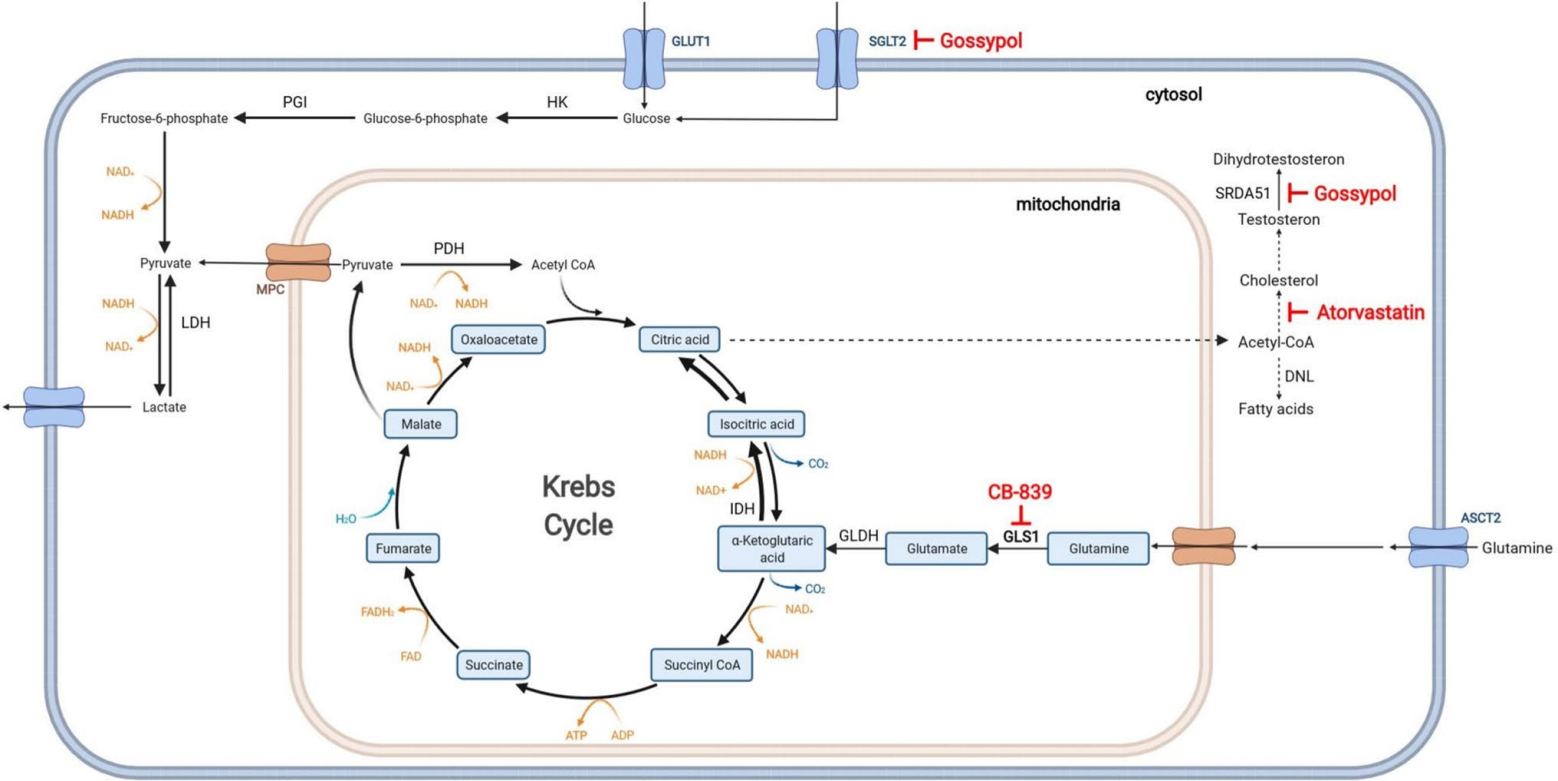
Overview of the glycolysis, glutaminolysis, and the Krebs cycle as well as therapeutic interventions in PCa. This diagram shows partial reactions of glycolysis, glutaminolysis, and the Krebs cycle. Orange arrows show the conversion of cofactors, the blue text indicates transporters, and the red text shows the target site of currently clinically tested inhibitors. Solid arrows represent single metabolic steps. Dashed arrows represent simplified multistep processes. HK (hexokinase), PGI (phosphoglucose isomerase), GLDH (glutamate dehydrogenase), GLS1 (glutaminase 1), GLUT1 (glucose transporter), ASCT2 (glutamine transporter), MPC (mitochondrial pyruvate carrier), SGLT2 (sodium-dependent glucose cotransporters), PDH (pyruvate dehydrogenase), and DNL (de novo lipogenesis)

Unlike other solid tumours, Gln plays a lesser role in low-risk primary PCa as lipids, succinate, and pyruvate seem to be the primary source of energy and biosynthesis (Fig. 2) (Bader et al. 2019; Flavin et al. 2011; Schöpf et al. 2020). However, metabolic reprogramming occurs during tumour progression, and Gln plays a more prominent role in advanced and aggressive PCa as well as CRPC (Kaushik et al. 2014; Sun et al. 2021a). The amino acid Gln is the most used source of anaplerosis (DeBerardinis et al. 2008). Although classified as a non-essential amino acid, Gln is the second most common extracellular nutrient and a key factor fuelling cancer cell growth (Li et al. 2021). In cancer cells, Gln is often called a semi-essential amino acid. The amino acid Gln is mainly, but not exclusively, transported by the alanine, serine, and cysteine-preferred transporter-2 (ASCT2) into the cell, which is expressed in benign and malignant prostate cells (Li et al. 2003). In addition, Gln provides a source of carbon and nitrogen groups for the Krebs cycle and NADPH for synthesising nucleotides, proteins, and lipids. Indeed, over the years, the aggressive behaviour of PCa cells has been linked with the use of glutaminolysis (Pan et al. 2015). Overall, CRPC cells display enhanced Gln metabolism sustained by the increased uptake of Gln and upregulated gene expression of the Gln metabolism key regulators glutaminase (GLS) 1 and 2 (Pan et al. 2015; Vayalil and Landar 2015). Gln can be converted to α-ketoglutarate (α-KG), thus maintaining the Krebs cycle (DeBerardinis et al. 2008). To this end, the mitochondrial GLS enzymes transform Gln into glutamate, which is converted to α-KG, a component utilised by the Krebs cycle (Li et al. 2021).

GLS has been reported to be a key enzyme in the Gln metabolism of cancer cells. Based on their primary expression in normal tissue, two different subtypes of glutaminase have been identified: GLS1, the kidney type and GLS2, the liver type. GLS1 is the isoenzyme predominantly expressed in PCa, and its expression levels are positively correlated with the tumour stage and PCa progression (Myint et al. 2021). In contrast, GLS2 has been reported to function as a tumour suppressor and is usually downregulated in cancer (Mates et al. 2018). The cancer-specific expression of GLS1 and the downregulation of GLS2 are based on the specific transcriptional regulation of the two proteins. The oncogene c-Myc was reported to increase GLS1 expression and promote tumour cell proliferation in PCa cells (Gao et al. 2009). In PCa cells, increased GLS1 expression via c-Myc is caused by post-transcriptional changes involving microRNAs miR-23a and miR-23b (Gao et al. 2009). On the other hand, the relationship between c-Myc and GLS1 is discussed controversially as the regulation seems to depend on the tumour entity. For example, GLS1 is downregulated in c-Myc-induced renal cell carcinoma, whereas GLS1 is upregulated in T-lymphocytes by c-Myc (Shroff et al. 2015; Wang et al. 2011).

GLS2 is primarily regulated by the transcription factor p53, which has a significantly high mutation frequency in PCa (~ 30–70%) (Ecke et al. 2010; Hu et al. 2010). However, next to the p53 family, it has been reported that two other transcription factors, p73 and p63, can control GLS2 expression during neuronal differentiation, epidermal differentiation, or tumourigenesis under high oxidative stress (Giacobbe et al. 2013; Velletri et al. 2013). In addition to c-Myc or p53, the isoenzymes are regulated cellularly depending on certain metabolic products. Thus, GLS1 is promoted by higher phosphate levels and inhibited by glutamate. In contrast, GLS2 is promoted by low phosphate levels and is not hindered by glutamate (Campos-Sandoval et al. 2007).

The metabolism can induce oxidative stress by high amounts of reactive oxygen species during tumour growth and proliferation. ROS molecules can serve as second messenger signalling, increasing inflammatory cytokines’ production and promoting PCa progression (Gupta-Elera et al. 2012). However, elevated ROS levels can damage biomolecules such as lipids, DNA, RNA, and proteins leading to dysfunction, inactivation, and apoptosis (Gupta-Elera et al. 2012). To protect biomolecules from oxidative stress, so-called antioxidant molecules such as glutathione are thus increasingly produced by tumour cells. Gln plays a vital role in the synthesis and regeneration of glutathione. On the other hand, Gln is used for synthesis on the one hand and provides reduced nitrogen for the molecule.

On the other hand, in localised PCa, Gln metabolism has shown to be critical in the development of radioresistance as the Gln-derived α-KG is crucial for glutathione production and ROS scavenging (Mukha et al. 2021). However, in CRPC, it has been shown that antioxidant activities are downregulated to increase ROS-mediated inflammatory cytokines production and promote CRPC progression and aggressiveness (Mondal et al. 2021). Therefore, Gln may not play a significant role in antioxidant activities in advanced PCa and CRPC.

DNL and cholesterol synthesis play an essential role in PCa metabolism as lipids serve as an energy source, membrane-building resources, and secondary messenger (Stoykova and Schlaepfer 2019). Several studies have revealed that citrate from the Krebs cycle can be involved in DNL and cholesterol synthesis (Ochoa Ruiz 2012). Gln is incorporated into the Krebs cycle after the deamination via GLS1 of Gln to glutamate. Glutamate is catalysed via several intermediate steps into citrate (Ochoa Ruiz 2012). Moreover, alterations in the isocitrate dehydrogenases (IDH) 1 and 2 proteins lead to direct catalysing of α-KG to citrate (Gonthier et al. 2019). Citrate can be exported into the cytoplasm, where the ATP citrate synthase can metabolise it into acetyl-CoA. Acetyl-CoA is later used for fatty acid and cholesterol synthesis. As cell-intrinsic DNL has been required for PCa cell proliferation and inhibition of Gln uptake by ASCT2 reducing DNL, Gln is likely to play a significant role in providing citrate mandatory for DNL (Chen et al. 2018; Wang et al. 2015).

Once in the cell, Gln can also be utilised as a nitrogen source for synthesising nucleotides (pyrimidines and purines) (Li et al. 2021). Therefore, Gln contributes to the de novo nucleotide synthesis, critical for DNA synthesis during cell proliferation and RNA synthesis during gene transcription. Direct involvement of Gln in nucleotide synthesis in PCa has not yet been demonstrated. However, due to the immense importance of nucleotides in many biological processes and cell division, involvement in this process of the de novo nucleotide synthesis in PCa is very likely.

### Metabolism in neuroendocrine PCa

The development of neuroendocrine prostate cancer (NEPC) is one of the biggest challenges in CRPC treatment (Aggarwal et al. 2018; Conteduca et al. 2019). In contrast to PCa, which generally represents an adenocarcinoma, NEPC is represented by small-cell neuroendocrine-like cells. However, only 40% are classified as pure small-cell carcinoma (Conteduca et al. 2019). NEPC displays an increased expression of typical neuroendocrine markers, such as synaptophysin, tubulin, beta 3 class III, tumour protein D52 isoform 1, and low AR and AR-target gene expression (Ather et al. 2008). In primary PCa, 2% of cells represent NEPC, whereas the incidence of NEPC is up to 30% after ADT (Aggarwal et al. 2018; Conteduca et al. 2019). External signals can initiate NEPC differentiation [calcitonin, serotonin, Interleukin (IL)-1 and IL-6] or dysregulation of internal signal pathways (e.g., cAMP response element-binding protein pathway or G-protein-coupled receptor kinase 3 pathway) (Diaz et al. 1998; Lin et al. 2017; Oelrich et al. 2021). As NEPC tumours diminish the AR signalling regulating many metabolic processes in PCa, activation of several signal pathways and epigenetic modifications cause metabolic reprogramming (Schvartzman et al. 2018). For example, increased activation and expression of EZH2 and histone lysine demethylase KDM8 reprogram NEPC metabolism towards aerobic glycolysis (Wang et al. 2019). The dependency on glycolysis could be validated by Choi SYC and colleagues, revealing reduced NEPC cell proliferation after targeting glucose metabolism in patient-derived xenograft models (Choi et al. 2018). As patients with NEPC treated with platinum-based therapies have a median progression-free survival of 3.9 months, targeting the metabolism may be an option to reduce the aggressive NEPC (Conteduca et al. 2019).

### Clinical trials targeting PCa’s metabolism

Maintaining anabolic metabolism is essential for tumour growth, presenting itself as an excellent opportunity for therapeutic intervention (Fig. 2). However, this therapy is particularly challenging, because metabolism-targeted treatments should selectively target the tumour while sparing healthy cells and tissues. Therefore, a study was initiated on men with symptomatic benign prostatic hyperplasia to investigate the effects of oral doses of lonidamine on prostate volume, prostate-specific antigen, and urine flow (Roehrborn 2005). The study was based on the mechanism of action of lonidamine, a derivate of indazole-3-carboxylic acid, disturbing the energy metabolism by interfering with glycolysis. Lonidamine treatment leads to a reduction of prostate volume in vivo (Heywood et al. 1981). Also, the first clinical trials revealed significant improvements in BPH symptoms and a significant decrease in prostate volume and PSA for at least 1 month after treatment, suggesting that lonidamine may be a therapeutic alternative for BPH (Ditonno et al. 2005). Despite these promising results, a randomised phase 3, double-blind, placebo-controlled study of lonidamine for treating BPH was terminated in 2009 (NCT00435448). A phase II study with lonidamine in patients with CRPC revealed that lonidamine had no benefits compared with standard therapies (Boccardo et al. 1992).

Only a few therapeutics targeting the prostate’s metabolism have been tested in clinical trials. The glucose inhibitor silybin has been tested in several discontinued clinical trials but stopped due to observed toxicities or low tissue accessibility (Flaig et al. 2010). Also, other glucose inhibitor strategies have been tested as therapeutic strategies. For example, 2-deoxyglucose is a glucose molecule that cannot be used in glycolysis and therefore inhibit the production of glucose-6-phosphate. First clinical data demonstrated promising results as 2-deoxyglucose induced autophagy in prostate cells (Stein et al. 2010). However, a phase I/II trial was stopped due to slow recruitment (NCT00633087).

In PCa, lipid metabolism (including DNL and cholesterol synthesis) has shown to be essential for multiple biological processes. The AR and ADT regulate genes involved in lipid metabolism and ADT mediates the downregulation of lipid metabolism during therapy (Butler et al. 2016). Due to reactivation of AR activity during CRPC development, reactivation of DNL also occurs. As described above, lipid metabolism promotes intratumoral androgen synthesis, a resistance mechanism to ADT. Different lipid metabolism inhibitors have now been tested for efficacy in preclinical and near-patient models to overcome this resistance mechanism. The cholesterol-lowering statin drug atorvastatin is currently being tested in a clinical phase 3 trial (NCT04026230). In the clinical trial, Atorvastatin is combined with ADT to delay therapy resistance caused by metabolic reprogramming and consequently postpone the development of CRPC (Siltari et al. 2022).

Another drug tested in clinical trials targeting PCa metabolism was Gossypol (AT-101). Gossypol targets the Na+ -dependent active glucose cellular transport and causes an uptake reduction of glucose, alanine, leucine, and calcium (Renner et al. 2022). Moreover, Gossypol has been described as an inhibitor of 5α-reductase 1, 3α-hydroxysteroid dehydrogenase, and retinol dehydrogenase 2, which are mandatory for androgen metabolism (Cao et al. 2019). In several phase 1/2 trials, Gossypol was tested alone or combined with ADT or docetaxel to treat patients with newly diagnosed metastatic PCa (NCT00666666, NCT00571675, NCT00286806, NCT00286793). Compared to ADT alone, Gossypol in combination with ADT did not increase the percentage of patients attaining biochemical relapse after 7.5 months of treatment (Stein et al. 2016). However, the combination leads to unexpected sensory neuropathy. Compared to the approved therapeutic docetaxel, a combination of docetaxel with Gossypol was tolerable, but did not extend overall survival (Sonpavde et al. 2012). As none of the studies met the prespecified activity level, these drug combinations were not further developed.

Due to its frequent alterations in tumours and malignant changes, glutamine metabolism has received particular attention and has been part of research for quite some time. In vitro studies demonstrated the relevance of GLS1 in promoting PCa cells’ survival and growth. GLS1 activity was related to increased cell viability, development, and invasiveness of PCa cells (Pan et al. 2015). Blocking GLS1, besides suppressing survival of PCa cells, also was suggested to affect glycolysis, decreasing ATP production and oxygen consumption, as well as glutathione production (Wang et al. 2013). Clinical testing of chemical inhibitors for GLS1 is part of current research. There are currently many clinical trials investigating the selective, reversible, and orally active inhibitor CB-839 in patients with advanced solid tumours (e.g., NCT02071862, NCT03875313, NCT02861300), showing encouraging clinical activity and tolerability. In addition, there is a planned phase 2 clinical trial for metastatic PCa (NCT04824937).

### Role of the metabolic crosstalk between the tumour microenvironment and PCa metabolism

This review focused on the changes in metabolism in normal and malignant prostate epithelial cells. However, an increasing number of studies revealed an essential role of the tumour microenvironment (TME) in tumour formation and progression in PCa (Dai et al. 2017). The TME describes the environment of PCa, including blood vessels, immune cells, fibroblasts, signalling molecules, and the extracellular matrix. Therefore, the TME is the source of signal proteins such as growth factors and cytokines (e.g., TGF-β, IL-4, IL-6, and IL-8), influencing PCa progression, androgen-independent conversion, and distal metastasis (Bonollo et al. 2020; Culig and Puhr 2018; Handle et al. 2018; Nappo et al. 2017; Niu and Xia 2009). Several studies report the influence of TME on PCa metabolism. Furthermore, cancer-associated fibroblasts (CAF) have established metabolic crosstalk with PCa epithelial cells and promote tumour progress and aggressiveness (Ippolito et al. 2019; Pértega-Gomes et al. 2014). Furthermore, cholesterol biosynthesis is overexpressed in advanced PCa and correlates with a bad prognosis (Kalogirou et al. 2021). Statins are potent inhibitors of the metabolic pathways producing cholesterol and are therefore also tested in several clinical trials in PCa (NCT04026230, NCT01561482, NCT01478828, NCT04094519). In a current study, the statin Atorvastatin’s effects are tested in combination with ADT (NCT04026230).

Furthermore, it is suggested that lactate secreted by CAFs alters the NAD+ /NADH ratio in the PCa cells, leading to an SIRT1-dependent PGC-1α activation (Ippolito et al. 2019). This PGC-1α activation enhances mitochondrial mass and activity, causing increased tumour growth. Until now, little is known about the metabolic crosstalk between the TME and PCa. However, there is an urgent need to work out the interaction between the TME and PCa metabolism to understand tumour progress, and develop novel therapeutic strategies targeting tumour metabolism.

## Conclusion

Reprogramming the cellular metabolism fuels the intense energy demands of fast-growing tumour cells. In this review, we focused on metabolic changes in the development and progress of PCa epithelial cells. In contrast to other solid cancers, early PCa gets primarily utilised by pyruvate and succinate. On the other hand, advanced stages and CRPC are fuelled by choline, amino acid, and glycolytic metabolism. Interestingly, cholesterol and lipid metabolism is essential in all PCa stages. However, when the metabolic reprogramming in PCa has been described well, the metabolic crosstalk between the TME and PCa epithelial cells is still not appropriately investigated. The lack of these findings could also explain why most clinical trials have not yet been able to stand up to established therapeutics. However, as the different stages of PCa seem to have unique metabolic features, the metabolism looks like an exceptional opportunity to develop tailored novel therapeutic strategies to synergise with established therapies.

## Data Availability

Not applicable.

## References

[CR1] Aggarwal R, Huang J, Alumkal JJ, Zhang L, Feng FY, Thomas GV, Weinstein AS, Friedl V, Zhang C, Witte ON, Lloyd P, Gleave M, Evans CP, Youngren J, Beer TM, Rettig M, Wong CK, True L, Foye A, Playdle D, Ryan CJ, Lara P, Chi KN, Uzunangelov V, Sokolov A, Newton Y, Beltran H, Demichelis F, Rubin MA, Stuart JM, Small EJ (2018). Clinical and genomic characterization of treatment-emergent small-cell neuroendocrine prostate cancer: a multi-institutional prospective study. J Clin Oncol.

[CR2] Ather MH, Abbas F, Faruqui N, Israr M, Pervez S (2008). Correlation of three immunohistochemically detected markers of neuroendocrine differentiation with clinical predictors of disease progression in prostate cancer. BMC Urol.

[CR3] Audet-Walsh E, Yee T, McGuirk S, Vernier M, Ouellet C, St-Pierre J, Giguere V (2017). Androgen-dependent repression of errgamma reprograms metabolism in prostate cancer. Cancer Res.

[CR4] Bader DA, Hartig SM, Putluri V, Foley C, Hamilton MP, Smith EA, Saha PK, Panigrahi A, Walker C, Zong L, Martini-Stoica H, Chen R, Rajapakshe K, Coarfa C, Sreekumar A, Mitsiades N, Bankson JA, Ittmann MM, O’Malley BW, Putluri N, McGuire SE (2019). Mitochondrial pyruvate import is a metabolic vulnerability in androgen receptor-driven prostate cancer. Nat Metab.

[CR5] Boccardo F, Guarneri D, Pace M, Decensi A, Oneto F, Martorana G (1992). Phase II study with lonidamine in the treatment of hormone-refractory prostatic cancer patients. Tumori.

[CR6] Bonollo F, Thalmann GN, Kruithof-de Julio M, Karkampouna S (2020). The role of cancer-associated fibroblasts in prostate cancer tumorigenesis. Cancers (basel).

[CR7] Butler LM, Centenera MM, Swinnen JV (2016). Androgen control of lipid metabolism in prostate cancer: novel insights and future applications. Endocr Relat Cancer.

[CR8] Cai H, Xu Z, Xu T, Yu B, Zou Q (2015). Diabetes mellitus is associated with elevated risk of mortality amongst patients with prostate cancer: a meta-analysis of 11 cohort studies. Diabetes Metab Res Rev.

[CR9] Campos-Sandoval JA, López de la Oliva AR, Lobo C, Segura JA, Matés JM, Alonso FJ, Márquez J (2007). Expression of functional human glutaminase in baculovirus system: affinity purification, kinetic and molecular characterization. Int J Biochem Cell Biol.

[CR10] Cao S, Wang G, Ge F, Li X, Zhu Q, Ge R-S, Wang Y (2019). Gossypol inhibits 5α-reductase 1 and 3α-hydroxysteroid dehydrogenase: its possible use for the treatment of prostate cancer. Fitoterapia.

[CR11] Chen J, Guccini I, Di Mitri D, Brina D, Revandkar A, Sarti M, Pasquini E, Alajati A, Pinton S, Losa M, Civenni G, Catapano CV, Sgrignani J, Cavalli A, D'Antuono R, Asara JM, Morandi A, Chiarugi P, Crotti S, Agostini M, Montopoli M, Masgras I, Rasola A, Garcia-Escudero R, Delaleu N, Rinaldi A, Bertoni F, Bono J, Carracedo A, Alimonti A (2018). Compartmentalized activities of the pyruvate dehydrogenase complex sustain lipogenesis in prostate cancer. Nat Genet.

[CR12] Choi SYC, Ettinger SL, Lin D, Xue H, Ci X, Nabavi N, Bell RH, Mo F, Gout PW, Fleshner NE, Gleave ME, Collins CC, Wang Y (2018). Targeting MCT4 to reduce lactic acid secretion and glycolysis for treatment of neuroendocrine prostate cancer. Cancer Med.

[CR13] Conteduca V, Oromendia C, Eng KW, Bareja R, Sigouros M, Molina A, Faltas BM, Sboner A, Mosquera JM, Elemento O, Nanus DM, Tagawa ST, Ballman KV, Beltran H (2019). Clinical features of neuroendocrine prostate cancer. Eur J Cancer.

[CR14] Cornford P, van den Bergh RCN, Briers E, Van den Broeck T, Cumberbatch MG, De Santis M, Fanti S, Fossati N, Gandaglia G, Gillessen S, Grivas N, Grummet J, Henry AM, der Kwast THV, Lam TB, Lardas M, Liew M, Mason MD, Moris L, Oprea-Lager DE, der Poel HGV, Rouvière O, Schoots IG, Tilki D, Wiegel T, Willemse PM, Mottet N (2021). EAU-EANM-ESTRO-ESUR-SIOG guidelines on prostate cancer. Part II-2020 update: treatment of relapsing and metastatic prostate cancer. Eur Urol.

[CR15] Costello LC, Franklin RB (1997). Citrate metabolism of normal and malignant prostate epithelial cells. Urology.

[CR16] Culig Z, Puhr M (2018). Interleukin-6 and prostate cancer: current developments and unsolved questions. Mol Cell Endocrinol.

[CR17] Dai J, Lu Y, Roca H, Keller JM, Zhang J, McCauley LK, Keller ET (2017). Immune mediators in the tumor microenvironment of prostate cancer. Chin J Cancer.

[CR18] DeBerardinis RJ, Lum JJ, Hatzivassiliou G, Thompson CB (2008). The biology of cancer: metabolic reprogramming fuels cell growth and proliferation. Cell Metab.

[CR19] Diaz M, Abdul M, Hoosein N (1998). Modulation of neuroendocrine differentiation in prostate cancer by interleukin-1 and -2. Prostate Suppl.

[CR20] Ditonno P, Battaglia M, Selvaggio O, Garofalo L, Lorusso V, Selvaggi FP (2005). Clinical evidence supporting the role of lonidamine for the treatment of BPH. Rev Urol.

[CR21] Ebersbach C, Beier A-MK, Thomas C, Erb HHH (2021). Impact of STAT proteins in tumor progress and therapy resistance in advanced and metastasised prostate cancer. Cancers.

[CR22] Ecke TH, Schlechte HH, Schiemenz K, Sachs MD, Lenk SV, Rudolph BD, Loening SA (2010). TP53 gene mutations in prostate cancer progression. Anticancer Res.

[CR23] Erb HHH, Bodenbender J, Handle F, Diehl T, Donix L, Tsaur I, Gleave M, Haferkamp A, Huber J, Fuessel S, Juengel E, Culig Z, Thomas C (2020). Assessment of STAT5 as a potential therapy target in enzalutamide-resistant prostate cancer. PLoS One.

[CR24] Ettinger SL, Sobel R, Whitmore TG, Akbari M, Bradley DR, Gleave ME, Nelson CC (2004). Dysregulation of sterol response element-binding proteins and downstream effectors in prostate cancer during progression to androgen independence. Cancer Res.

[CR25] Fair WR, Cordonnier JJ (1977). The pH of prostatic fluid: a reappraisal and therapeutic implications. Trans Am Assoc Genitourin Surg.

[CR26] Ferlay J, Colombet M, Soerjomataram I, Parkin DM, Pineros M, Znaor A, Bray F (2021). Cancer statistics for the year 2020: an overview. Int J Cancer.

[CR27] Flaig TW, Glodé M, Gustafson D, van Bokhoven A, Tao Y, Wilson S, Su LJ, Li Y, Harrison G, Agarwal R, Crawford ED, Lucia MS, Pollak M (2010). A study of high-dose oral silybin-phytosome followed by prostatectomy in patients with localised prostate cancer. Prostate.

[CR28] Flavin R, Zadra G, Loda M (2011). Metabolic alterations and targeted therapies in prostate cancer. J Pathol.

[CR29] Gao P, Tchernyshyov I, Chang TC, Lee YS, Kita K, Ochi T, Zeller KI, De Marzo AM, Van Eyk JE, Mendell JT, Dang CV (2009). c-Myc suppression of miR-23a/b enhances mitochondrial glutaminase expression and glutamine metabolism. Nature.

[CR30] Giacobbe A, Bongiorno-Borbone L, Bernassola F, Terrinoni A, Markert EK, Levine AJ, Feng Z, Agostini M, Zolla L, Agrò AF, Notterman DA, Melino G, Peschiaroli A (2013). p63 regulates glutaminase 2 expression. Cell Cycle.

[CR31] Gonthier K, Poluri RTK, Weidmann C, Tadros M, Audet-Walsh É (2019). Reprogramming of Isocitrate dehydrogenases expression and activity by the androgen receptor in prostate cancer. Mol Cancer Res.

[CR32] Gupta-Elera G, Garrett AR, Robison RA, O'Neill KL (2012). The role of oxidative stress in prostate cancer. Eur J Cancer Prev.

[CR33] Han W, Gao S, Barrett D, Ahmed M, Han D, Macoska JA, He HH, Cai C (2018). Reactivation of androgen receptor-regulated lipid biosynthesis drives the progression of castration-resistant prostate cancer. Oncogene.

[CR34] Hanahan D, Weinberg RA (2011). Hallmarks of cancer: the next generation. Cell.

[CR35] Handle F, Puhr M, Schaefer G, Lorito N, Hoefer J, Gruber M, Guggenberger F, Santer FR, Marques RB, van Weerden WM, Claessens F, Erb HHH, Culig Z (2018). The STAT3 inhibitor galiellalactone reduces IL6-mediated AR activity in benign and malignant prostate models. Mol Cancer Ther.

[CR36] Hennequin C, Hannoun-Lévi JM, Rozet F (2017). Management of local relapse after prostate cancer radiotherapy: surgery or radiotherapy?. Cancer Radiother.

[CR37] Hensley CT, Wasti AT, DeBerardinis RJ (2013). Glutamine and cancer: cell biology, physiology, and clinical opportunities. J Clin Invest.

[CR38] Heywood R, James RW, Barcellona PS, Campana A, Cioli V (1981). Toxicological studies on 1-substituted-indazole-3-carboxylic acids. Chemotherapy.

[CR39] Hu W, Zhang C, Wu R, Sun Y, Levine A, Feng Z (2010). Glutaminase 2, a novel p53 target gene regulating energy metabolism and antioxidant function. Proc Natl Acad Sci U S A.

[CR40] Ippolito L, Morandi A, Taddei ML, Parri M, Comito G, Iscaro A, Raspollini MR, Magherini F, Rapizzi E, Masquelier J, Muccioli GG, Sonveaux P, Chiarugi P, Giannoni E (2019). Cancer-associated fibroblasts promote prostate cancer malignancy via metabolic rewiring and mitochondrial transfer. Oncogene.

[CR41] Kalogirou C, Linxweiler J, Schmucker P, Snaebjornsson MT, Schmitz W, Wach S, Krebs M, Hartmann E, Puhr M, Müller A, Spahn M, Seitz AK, Frank T, Marouf H, Büchel G, Eckstein M, Kübler H, Eilers M, Saar M, Junker K, Röhrig F, Kneitz B, Rosenfeldt MT, Schulze A (2021). MiR-205-driven downregulation of cholesterol biosynthesis through SQLE-inhibition identifies therapeutic vulnerability in aggressive prostate cancer. Nat Commun.

[CR42] Kaushik AK, Vareed SK, Basu S, Putluri V, Putluri N, Panzitt K, Brennan CA, Chinnaiyan AM, Vergara IA, Erho N, Weigel NL, Mitsiades N, Shojaie A, Palapattu G, Michailidis G, Sreekumar A (2014). Metabolomic profiling identifies biochemical pathways associated with castration-resistant prostate cancer. J Proteome Res.

[CR43] Lee J, Giovannucci E, Jeon JY (2016). Diabetes and mortality in patients with prostate cancer: a meta-analysis. Springerplus.

[CR44] Li R, Younes M, Frolov A, Wheeler TM, Scardino P, Ohori M, Ayala G (2003). Expression of neutral amino acid transporter ASCT2 in human prostate. Anticancer Res.

[CR45] Li T, Copeland C, Le A (2021). Glutamine metabolism in cancer. Adv Exp Med Biol.

[CR46] Lin LC, Gao AC, Lai CH, Hsieh JT, Lin H (2017). Induction of neuroendocrine differentiation in castration resistant prostate cancer cells by adipocyte differentiation-related protein (ADRP) delivered by exosomes. Cancer Lett.

[CR47] Liu IJ, Zafar MB, Lai YH, Segall GM, Terris MK (2001). Fluorodeoxyglucose positron emission tomography studies in diagnosis and staging of clinically organ-confined prostate cancer. Urology.

[CR48] Lloyd MD, Darley DJ, Wierzbicki AS, Threadgill MD (2008). α-Methylacyl-CoA racemase – an ‘obscure’ metabolic enzyme takes centre stage. FEBS J.

[CR49] Lounis MA, Peant B, Leclerc-Desaulniers K, Ganguli D, Daneault C, Ruiz M, Zoubeidi A, Mes-Masson AM, Saad F (2020). Modulation of de novo lipogenesis improves response to enzalutamide treatment in prostate cancer. Cancers (basel).

[CR50] Mates JM, Campos-Sandoval JA, Marquez J (2018). Glutaminase isoenzymes in the metabolic therapy of cancer. Biochim Biophys Acta Rev Cancer.

[CR51] Migita T, Ruiz S, Fornari A, Fiorentino M, Priolo C, Zadra G, Inazuka F, Grisanzio C, Palescandolo E, Shin E, Fiore C, Xie W, Kung AL, Febbo PG, Subramanian A, Mucci L, Ma J, Signoretti S, Stampfer M, Hahn WC, Finn S, Loda M (2009). Fatty acid synthase: a metabolic enzyme and candidate oncogene in prostate cancer. J Natl Cancer Inst.

[CR52] Mondal D, Narwani D, Notta S, Ghaffar D, Mardhekar N, Quadri SSA (2021). Oxidative stress and redox signaling in CRPC progression: therapeutic potential of clinically-tested Nrf2-activators. Cancer Drug Resist.

[CR53] Montgomery RB, Mostaghel EA, Vessella R, Hess DL, Kalhorn TF, Higano CS, True LD, Nelson PS (2008). Maintenance of intratumoral androgens in metastatic prostate cancer: a mechanism for castration-resistant tumor growth. Cancer Res.

[CR54] Mottet N, van den Bergh RCN, Briers E, Van den Broeck T, Cumberbatch MG, De Santis M, Fanti S, Fossati N, Gandaglia G, Gillessen S, Grivas N, Grummet J, Henry AM, van der Kwast TH, Lam TB, Lardas M, Liew M, Mason MD, Moris L, Oprea-Lager DE, van der Poel HG, Rouviere O, Schoots IG, Tilki D, Wiegel T, Willemse PM, Cornford P (2021). EAU-EANM-ESTRO-ESUR-SIOG guidelines on prostate cancer-2020 update. Part 1: screening, diagnosis, and local treatment with curative intent. Eur Urol.

[CR55] Mukha A, Kahya U, Linge A, Chen O, Löck S, Lukiyanchuk V, Richter S, Alves TC, Peitzsch M, Telychko V, Skvortsov S, Negro G, Aschenbrenner B, Skvortsova I-I, Mirtschink P, Lohaus F, Hölscher T, Neubauer H, Rivandi M, Labitzky V, Lange T, Franken A, Behrens B, Stoecklein NH, Toma M, Sommer U, Zschaeck S, Rehm M, Eisenhofer G, Schwager C, Abdollahi A, Groeben C, Kunz-Schughart LA, Baretton GB, Baumann M, Krause M, Peitzsch C, Dubrovska A (2021). GLS-driven glutamine catabolism contributes to prostate cancer radiosensitivity by regulating the redox state, stemness and ATG5-mediated autophagy. Theranostics.

[CR56] Myint ZW, Sun RC, Hensley PJ, James AC, Wang P, Strup SE, McDonald RJ, Yan D, St. Clair WH, Allison DB (2021). Evaluation of glutaminase expression in prostate adenocarcinoma and correlation with clinicopathologic parameters. Cancers.

[CR57] Nappo G, Handle F, Santer FR, McNeill RV, Seed RI, Collins AT, Morrone G, Culig Z, Maitland NJ, Erb HHH (2017). The immunosuppressive cytokine interleukin-4 increases the clonogenic potential of prostate stem-like cells by activation of STAT6 signalling. Oncogenesis.

[CR58] Niu Y-N, Xia S-J (2009). Stroma-epithelium crosstalk in prostate cancer. Asian J Androl.

[CR59] Obinata D, Lawrence MG, Takayama K, Choo N, Risbridger GP, Takahashi S, Inoue S (2020). Recent discoveries in the androgen receptor pathway in castration-resistant prostate cancer. Front Oncol.

[CR60] Ochoa Ruiz E (2012). Anaplerosis in cancer: another step beyond the warburg effect. Am J Mol Biol.

[CR61] Oczkowski M, Dziendzikowska K, Pasternak-Winiarska A, Wlodarek D, Gromadzka-Ostrowska J (2021). Dietary factors and prostate cancer development, progression, and reduction. Nutrients.

[CR62] Oelrich F, Junker H, Stope MB, Erb HHH, Walther R, Venz S, Zimmermann U (2021). Gelsolin governs the neuroendocrine transdifferentiation of prostate cancer cells and suppresses the apoptotic machinery. Anticancer Res.

[CR63] Owen DH, Katz DF (2005). A review of the physical and chemical properties of human semen and the formulation of a semen simulant. J Androl.

[CR64] Palermo G, Foschi N, D'Agostino D, Sacco E, Bassi P, Pinto F (2016). Local relapse of prostate cancer after primary definitive treatment: the management. Minerva Urol Nefrol.

[CR65] Pan T, Gao L, Wu G, Shen G, Xie S, Wen H, Yang J, Zhou Y, Tu Z, Qian W (2015). Elevated expression of glutaminase confers glucose utilisation via glutaminolysis in prostate cancer. Biochem Biophys Res Commun.

[CR66] Pavlova NN, Zhu J, Thompson CB (2022). The hallmarks of cancer metabolism: still emerging. Cell Metab.

[CR67] Pértega-Gomes N, Vizcaíno JR, Attig J, Jurmeister S, Lopes C, Baltazar F (2014). A lactate shuttle system between tumour and stromal cells is associated with poor prognosis in prostate cancer. BMC Cancer.

[CR68] Petros JA, Baumann AK, Ruiz-Pesini E, Amin MB, Sun CQ, Hall J, Lim S, Issa MM, Flanders WD, Hosseini SH, Marshall FF, Wallace DC (2005). mtDNA mutations increase tumorigenicity in prostate cancer. Proc Natl Acad Sci U S A.

[CR69] Renner O, Mayer M, Leischner C, Burkard M, Berger A, Lauer UM, Venturelli S, Bischoff SC (2022). Systematic review of Gossypol/AT-101 in cancer clinical trials. Pharmaceuticals (basel).

[CR70] Roehrborn CG (2005). The development of lonidamine for benign prostatic hyperplasia and other indications. Rev Urol.

[CR71] Sadeghi RN, Karami-Tehrani F, Salami S (2015). Targeting prostate cancer cell metabolism: impact of hexokinase and CPT-1 enzymes. Tumour Biol.

[CR72] Scaglia N, Frontini-López YR, Zadra G (2021). Prostate cancer progression: as a matter of fats. Front Oncol.

[CR73] Schiliro C, Firestein BL (2021). Mechanisms of metabolic reprogramming in cancer cells supporting enhanced growth and proliferation. Cells.

[CR74] Schöpf B, Weissensteiner H, Schäfer G, Fazzini F, Charoentong P, Naschberger A, Rupp B, Fendt L, Bukur V, Giese I, Sorn P, Sant’Anna-Silva AC, Iglesias-Gonzalez J, Sahin U, Kronenberg F, Gnaiger E, Klocker H,  (2020). OXPHOS remodeling in high-grade prostate cancer involves mtDNA mutations and increased succinate oxidation. Nat Commun.

[CR75] Schvartzman JM, Thompson CB, Finley LWS (2018). Metabolic regulation of chromatin modifications and gene expression. J Cell Biol.

[CR76] Shroff EH, Eberlin LS, Dang VM, Gouw AM, Gabay M, Adam SJ, Bellovin DI, Tran PT, Philbrick WM, Garcia-Ocana A, Casey SC, Li Y, Dang CV, Zare RN, Felsher DW (2015). MYC oncogene overexpression drives renal cell carcinoma in a mouse model through glutamine metabolism. Proc Natl Acad Sci U S A.

[CR77] Siegel DA, O'Neil ME, Richards TB, Dowling NF, Weir HK (2020). Prostate cancer incidence and survival, by stage and race/ethnicity - United States, 2001–2017. MMWR Morb Mortal Wkly Rep.

[CR78] Siltari A, Riikonen J, Koskimäki J, Pakarainen T, Ettala O, Boström P, Seikkula H, Kotsar A, Tammela T, Helminen M, Raittinen PV, Lehtimäki T, Fode M, Østergren P, Borre M, Rannikko A, Marttila T, Salonen A, Ronkainen H, Löffeler S, Murtola TJ (2022). Randomised double-blind phase 3 clinical study testing impact of atorvastatin on prostate cancer progression after initiation of androgen deprivation therapy: study protocol. BMJ Open.

[CR79] Singh KK, Desouki MM, Franklin RB, Costello LC (2006). Mitochondrial aconitase and citrate metabolism in malignant and nonmalignant human prostate tissues. Mol Cancer.

[CR80] Sonpavde G, Matveev V, Burke JM, Caton JR, Fleming MT, Hutson TE, Galsky MD, Berry WR, Karlov P, Holmlund JT, Wood BA, Brookes M, Leopold L (2012). Randomised phase II trial of docetaxel plus prednisone in combination with placebo or AT-101, an oral small molecule Bcl-2 family antagonist, as first-line therapy for metastatic castration-resistant prostate cancer. Ann Oncol.

[CR81] Stein M, Lin H, Jeyamohan C, Dvorzhinski D, Gounder M, Bray K, Eddy S, Goodin S, White E, Dipaola RS (2010). Targeting tumor metabolism with 2-deoxyglucose in patients with castrate-resistant prostate cancer and advanced malignancies. Prostate.

[CR82] Stein MN, Hussain M, Stadler WM, Liu G, Tereshchenko IV, Goodin S, Jeyamohan C, Kaufman HL, Mehnert J, DiPaola RS (2016). A phase II study of AT-101 to overcome Bcl-2–mediated resistance to androgen deprivation therapy in patients with newly diagnosed castration-sensitive metastatic prostate cancer. Clin Genitourin Cancer.

[CR83] Stoykova GE, Schlaepfer IR (2019). Lipid metabolism and endocrine resistance in prostate cancer, and new opportunities for therapy. Int J Mol Sci.

[CR84] Sun J, Bok RA, DeLos SJ, Upadhyay D, DeLos SR, Agarwal S, Van Criekinge M, Vigneron DB, Aggarwal R, Peehl DM, Kurhanewicz J, Sriram R (2021). Resistance to androgen deprivation leads to altered metabolism in human and murine prostate cancer cell and tumor models. Metabolites.

[CR85] Sun RF, Zhao CY, Chen S, Yu W, Zhou MM, Gao CR (2021). Androgen receptor stimulates hexokinase 2 and induces glycolysis by PKA/CREB signaling in hepatocellular carcinoma. Dig Dis Sci.

[CR86] Sung H, Ferlay J, Siegel RL, Laversanne M, Soerjomataram I, Jemal A, Bray F (2021). Global cancer statistics 2020: GLOBOCAN estimates of incidence and mortality worldwide for 36 cancers in 185 countries. CA Cancer J Clin.

[CR87] Swinnen JV, Van Veldhoven PP, Esquenet M, Heyns W, Verhoeven G (1996). Androgens markedly stimulate the accumulation of neutral lipids in the human prostatic adenocarcinoma cell line LNCaP. Endocrinology.

[CR88] Tan K, Naylor MJ (2022). The influence of modifiable factors on breast and prostate cancer risk and disease progression. Front Physiol.

[CR89] Tsouko E, Khan AS, White MA, Han JJ, Shi Y, Merchant FA, Sharpe MA, Xin L, Frigo DE (2014). Regulation of the pentose phosphate pathway by an androgen receptor-mTOR-mediated mechanism and its role in prostate cancer cell growth. Oncogenesis.

[CR90] Uo T, Sprenger CC, Plymate SR (2020). Androgen receptor signaling and metabolic and cellular plasticity during progression to castration resistant prostate cancer. Front Oncol.

[CR91] Vaupel P, Multhoff G (2021). Revisiting the Warburg effect: historical dogma versus current understanding. J Physiol.

[CR92] Vayalil PK, Landar A (2015). Mitochondrial oncobioenergetic index: a potential biomarker to predict progression from indolent to aggressive prostate cancer. Oncotarget.

[CR93] Velletri T, Romeo F, Tucci P, Peschiaroli A, Annicchiarico-Petruzzelli M, Niklison-Chirou MV, Amelio I, Knight RA, Mak TW, Melino G, Agostini M (2013). GLS2 is transcriptionally regulated by p73 and contributes to neuronal differentiation. Cell Cycle.

[CR94] Wang R, Dillon CP, Shi LZ, Milasta S, Carter R, Finkelstein D, McCormick LL, Fitzgerald P, Chi H, Munger J, Green DR (2011). The transcription factor Myc controls metabolic reprogramming upon T lymphocyte activation. Immunity.

[CR95] Wang Q, Tiffen J, Bailey CG, Lehman ML, Ritchie W, Fazli L, Metierre C, Feng YJ, Li E, Gleave M, Buchanan G, Nelson CC, Rasko JE, Holst J (2013). Targeting amino acid transport in metastatic castration-resistant prostate cancer: effects on cell cycle, cell growth, and tumor development. J Natl Cancer Inst.

[CR96] Wang Q, Hardie R-A, Hoy AJ, van Geldermalsen M, Gao D, Fazli L, Sadowski MC, Balaban S, Schreuder M, Nagarajah R, Wong JJL, Metierre C, Pinello N, Otte NJ, Lehman ML, Gleave M, Nelson CC, Bailey CG, Ritchie W, Rasko JEJ, Holst J (2015). Targeting ASCT2-mediated glutamine uptake blocks prostate cancer growth and tumour development. J Pathol.

[CR97] Wang HJ, Pochampalli M, Wang LY, Zou JX, Li PS, Hsu SC, Wang BJ, Huang SH, Yang P, Yang JC, Chu CY, Hsieh CL, Sung SY, Li CF, Tepper CG, Ann DK, Gao AC, Evans CP, Izumiya Y, Chuu CP, Wang WC, Chen HW, Kung HJ (2019). KDM8/JMJD5 as a dual coactivator of AR and PKM2 integrates AR/EZH2 network and tumor metabolism in CRPC. Oncogene.

[CR98] Wang J, Xu W, Wang B, Lin G, Wei Y, Abudurexiti M, Zhu W, Liu C, Qin X, Dai B, Wan F, Zhang H, Zhu Y, Ye D (2020). GLUT1 is an AR target contributing to tumor growth and glycolysis in castration-resistant and enzalutamide-resistant prostate cancers. Cancer Lett.

[CR99] White MA, Tsouko E, Lin C, Rajapakshe K, Spencer JM, Wilkenfeld SR, Vakili SS, Pulliam TL, Awad D, Nikolos F, Katreddy RR, Kaipparettu BA, Sreekumar A, Zhang X, Cheung E, Coarfa C, Frigo DE (2018). GLUT12 promotes prostate cancer cell growth and is regulated by androgens and CaMKK2 signaling. Endocr Relat Cancer.

[CR100] Xu M, Sakamoto S, Matsushima J, Kimura T, Ueda T, Mizokami A, Kanai Y, Ichikawa T (2016). Up-regulation of LAT1 during antiandrogen therapy contributes to progression in prostate cancer cells. J Urol.

[CR101] Xu L, Ma E, Zeng T, Zhao R, Tao Y, Chen X, Groth J, Liang C, Hu H, Huang J (2019). ATM deficiency promotes progression of CRPC by enhancing Warburg effect. Endocr Relat Cancer.

[CR102] Xu Z, Huang L, Dai T, Pei X, Xia L, Zeng G, Ye M, Liu K, Zeng F, Han W, Jiang S (2021). SQLE mediates metabolic reprogramming to promote LN metastasis in castration-resistant prostate cancer. Onco Targets Ther.

[CR103] Zheng H, Zhu Y, Shao X, Cai A, Dong B, Xue W, Gao H (2020). Distinct metabolic signatures of hormone-sensitive and castration-resistant prostate cancer revealed by a 1H NMR-based metabolomics of biopsy tissue. J Proteome Res.

[CR104] Zhu J, Thompson CB (2019). Metabolic regulation of cell growth and proliferation. Nat Rev Mol Cell Biol.

